# Modular bioengineering of whole-cell catalysis for sialo-oligosaccharide production: coordinated co-expression of CMP-sialic acid synthetase and sialyltransferase

**DOI:** 10.1186/s12934-023-02249-1

**Published:** 2023-11-27

**Authors:** Sabine Schelch, Manuel Eibinger, Jasmin Zuson, Jürgen Kuballa, Bernd Nidetzky

**Affiliations:** 1 Austrian Centre of Industrial Biotechnology, Krenngasse 37, 8010 Graz, Austria; 2 Institute of Biotechnology and Biochemical Engineering, Graz University of Technology, NAWI Graz, Petersgasse 12, 8010 Graz, Austria; 3 GALAB Laboratories GmbH, Am Schleusengraben 7, 21029 Hamburg, Germany

**Keywords:** Sialo-oligosaccharides, 3ʹ-Sialyllactose, α2,3‐Sialyltransferase, Whole-cell bio-catalysis, Multienzyme cascade reaction, Co-expression

## Abstract

**Background:**

In whole-cell bio-catalysis, the biosystems engineering paradigm shifts from the global reconfiguration of cellular metabolism as in fermentation to a more focused, and more easily modularized, optimization of comparably short cascade reactions. Human milk oligosaccharides (HMO) constitute an important field for the synthetic application of cascade bio-catalysis in resting or non-living cells. Here, we analyzed the central catalytic module for synthesis of HMO-type sialo-oligosaccharides, comprised of CMP-sialic acid synthetase (CSS) and sialyltransferase (SiaT), with the specific aim of coordinated enzyme co-expression in *E. coli* for reaction flux optimization in whole cell conversions producing 3′-sialyllactose (3SL).

**Results:**

Difference in enzyme specific activity (CSS from *Neisseria meningitidis*: 36 U/mg; α2,3-SiaT from *Pasteurella dagmatis*: 5.7 U/mg) was compensated by differential protein co-expression from tailored plasmid constructs, giving balance between the individual activities at a high level of both (α2,3-SiaT: 9.4 × 10^2^ U/g cell dry mass; CSS: 3.4 × 10^2^ U/g cell dry mass). Finally, plasmid selection was guided by kinetic modeling of the coupled CSS-SiaT reactions in combination with comprehensive analytical tracking of the multistep conversion (lactose, *N*-acetyl neuraminic acid (Neu5Ac), cytidine 5′-triphosphate; each up to 100 mM). The half-life of SiaT in permeabilized cells (≤ 4 h) determined the efficiency of 3SL production at 37 °C. Reaction at 25 °C gave 3SL (40 ± 4 g/L) in ∼ 70% yield within 3 h, reaching a cell dry mass-specific productivity of ∼ 3 g/(g h) and avoiding intermediary CMP-Neu5Ac accumulation.

**Conclusions:**

Collectively, balanced co-expression of CSS and SiaT yields an efficient (high-flux) sialylation module to support flexible development of *E. coli* whole-cell catalysts for sialo-oligosaccharide production.

**Supplementary Information:**

The online version contains supplementary material available at 10.1186/s12934-023-02249-1.

## Background

Biological systems engineering is an important discipline of bioengineering used for cell factory development. In a broad sense, its approach is to reconfigure the cellular metabolism with the aim of improving the performance of the growing cell in bioproduction [1, 2]. Whole-cell bio-catalysis, by contrast, often applies the cells in a growth-arrested state or uses them in a pretreated (e.g., permeabilized), hence no longer living form [3]. The paradigm shifts from the global perspective on metabolism to the more focused analysis of comparably shorter cascade reactions [3, 4]. The task of the biological conversion shifts from the balanced metabolization of nutrients in different biochemical pathways to the transformation of a specific set of substrates in precisely defined sequences of enzymatic steps [5–8]. Provided that the substrates used meet the demands of a viable production process, whole-cell bio-catalysis presents an attractive option for the process development [3, 4]. Subdivision of enzyme cascades into compact functional modules offers flexibility for rapid prototyping of whole-cell catalysts [9–13]. Compared to fermentation, realization of the bioconversion as decoupled from the cell growth opens up a larger space of engineering parameters (e.g., catalyst loading) amenable to process intensification and optimization [14, 15].

**Fig. 1:**
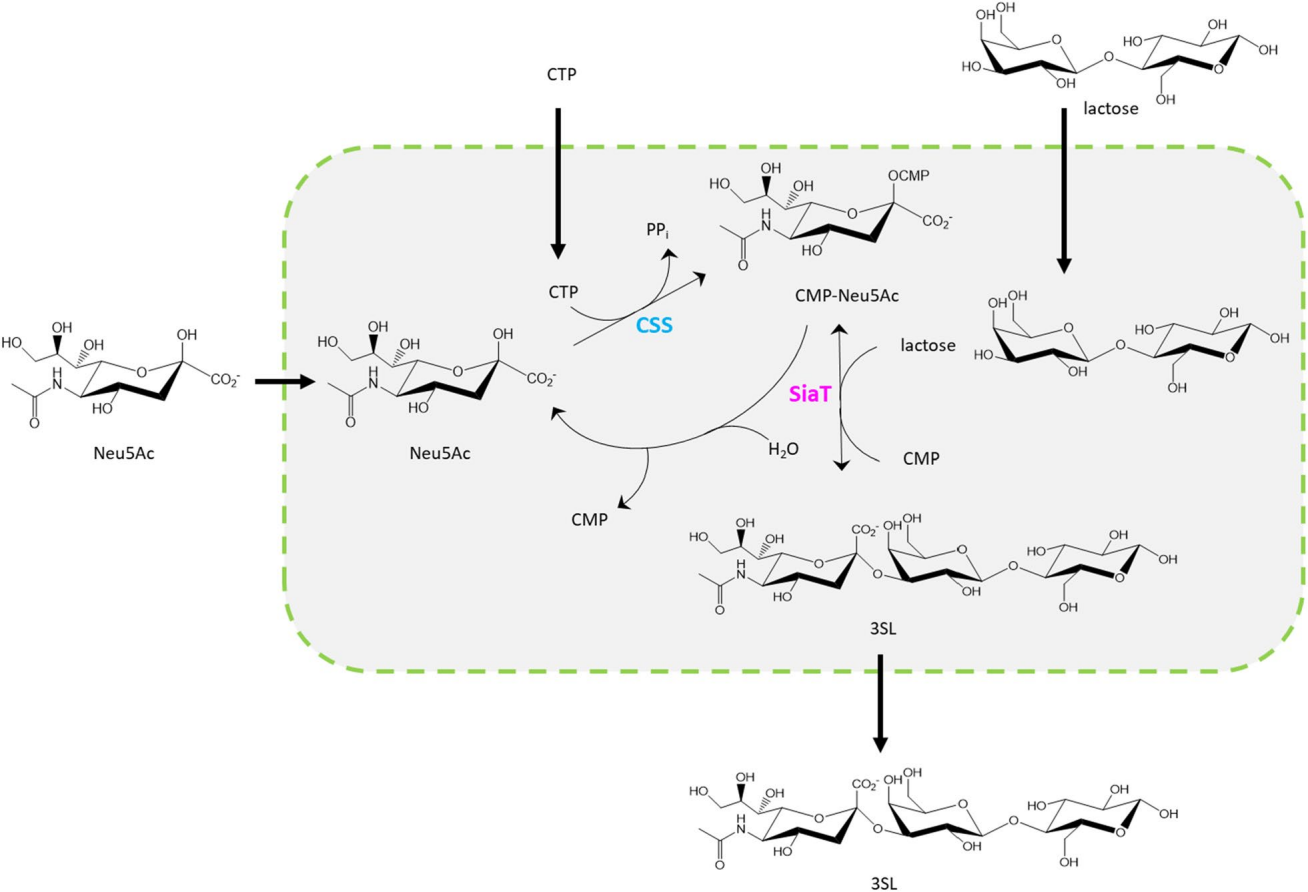
3SL synthesis by coupled reactions of CSS and α2,3-SiaT. The α2,3-SiaT is shown to also hydrolyze CMP-Neu5Ac in a side reaction. *CSS* CMP-sialic acid synthetase, *SiaT* sialyltransferase

Human milk oligosaccharides (HMOs) have attracted significant interest for use as pre-biotic ingredients [16, 17]. Industrial-scale bioproduction is promising to supply HMOs to emerging markets in health-related food [18, 19]. About 10–20% of the HMOs contain sialic acid, usually *N*-acetyl neuraminic acid (Neu5Ac), attached to an oligosaccharide core [20]. The most abundant of the sialo-oligosaccharides (2–6% of all HMOs) is sialo-lactose (SL) [21]. SL is an important target for production by whole-cell bio-catalysis [22–26]. Proposed synthetic routes involve stereo- and regioselective glycosylation of unprotected lactose (supplied directly as substrate) as the key step [27–30]. Sialyltransferase (SiaT) exhibits the required selectivity, with exceptions discussed later, and is arguably the most suitable candidate enzyme with which to perform sialylation [28]. SiaT uses cytidine 5’-monophosphate (CMP)-activated Neu5Ac (CMP-Neu5Ac) for activity (Fig. 1). SiaT applicability for synthesis relies on cost-efficient supply of CMP-Neu5Ac to the transformation [27, 30, 31]. This is typically realized by coupling the SiaT reaction to auxiliary reactions for CMP-Neu5Ac release in situ [28]. Considering that Neu5Ac can be produced efficiently from nutrients (e.g., glycerol) using engineered microbial strains [32–34], a parsimonious cascade reaction for SL synthesis is that of Fig. 1. Neu5Ac is considered here to be a usable starting material.

Sialylation is achieved in an atom-efficient transformation. Modular approach to the development of a whole-cell catalyst can involve functional subdivision of the cascade into the reactions of the actual synthesis (sialylation module) and the provision of chemical energy for CMP-Neu5Ac formation (CTP module). The subdivision into modules follows the idea that each module must exhibit a certain degree of functional independence of the respective other module. The sialylation module exhibits internal integration due enzymatic reactions linked to CMP-Neu5Ac. It is connected to the CTP module by the CTP used and the CMP produced. However, the CTP recycling from CMP is well established from earlier works, done with isolated enzymes in vitro [35–39] or in whole cells [26, 40]. The current study was therefore focused on the sialylation module which is less well explored. Its goal was to develop an efficient two-step cascade sialylation in *E. coli* based on optimized co-expression of CMP-Neu5Ac synthetase (CSS) and SiaT. Co-expression of CSS and SiaT was used previously in cell engineering for sialo-oligosaccharide synthesis by fermentation [22–24] or whole cell catalysis [26, 40]. However, the two enzymes were generally used just as they were received in the cells, without attention paid to their actual activities and the activity ratio. Li et al. mixed *E. coli* cell extracts containing individual CSS (from *Neisseria meningitidis*) and SiaT (from *N. gonorrhoeae*) and showed that change in the enzyme ratio (activities were not determined) had strong effect on the sialo-oligosaccharide release [39]. Enzyme co-expression in a single cell is evidently more practical than preparing each enzyme individually.

Sialylation module development involves the metabolic engineering challenge of optimizing the sialyl residue flux, given that CSS (the enzyme catalyzing the first step) exhibits higher (~ sixfold) specific activity than SiaT [41]. In a whole cell co-expressing both enzymes, therefore, differential protein expression is required to compensate imbalance in activities otherwise resulting [42]. When CSS activity is present in excess, CMP-Neu5Ac will accumulate as intermediate. The overall specific rate of 3SL formation (normalized on the total amount of CSS and SiaT protein) will thus be lower than it could be if the activities were balanced. CMP-Neu5Ac is also vulnerable to chemical decomposition [43, 44]. Avoidance of rate limitation by the SiaT step, to prevent loss of intermediary CMP-Neu5Ac, can thus benefit the product yield. A simple strategy that is often applied to balance the activities of multiple enzymes is to down-regulate the expression of the most active enzyme. Downregulating individual activities usually comes at the cost of a reduced overall (mass-specific) activity of the whole-cell catalyst [8, 45, 46]. A more promising strategy, yet one that is more challenging to realize, is to balance the individual activities at a high level of both [42, 45, 47]. CSS and SiaT both involve two-substrate reactions, with rates depending on the substrate concentrations in the usual (hyperbolic) way [41]. The overall rate as composite of the individual rates involves interconnected dependence on enzyme and substrate concentrations, rendering its optimization by the relative levels of protein co-expression a complex task. Additionally, the α2,3-SiaT from *Pasteurella dagmatis* used here [41], and other related SiaT enzymes from bacterial sources [21, 28], catalyze CMP-Neu5Ac hydrolysis in small degree (≤ 15%) next to sialyl transfer to the acceptor. The slightly imperfect reaction selectivity of the α2,3-SiaT affects the overall conversion in both rate and yield [41, 48]. Lastly, while the CSS reaction is effectively irreversible, the equilibrium position of the α2,3-SiaT reaction is relevant in a fully optimized transformation [41].

To include the multiple interconnected factors of reaction efficiency in the development of a whole cell catalyst, we used a kinetic model of the reactions by coupled CSS and α2,3-SiaT [41]. The model was essential to select the best construct as it provides guidance for the fine-tuned balancing of activities during the enzyme co-expression. Kinetic model prediction was also instrumental to identify enzyme inactivation as an unanticipated factor of conversion efficiency. Different genetic designs of plasmid vector [42] were explored to eventually obtain an *E. coli* whole cell catalyst that featured high activity of both enzymes at a suitable ratio α2,3-SiaT:CSS ≥ 1 (α2,3-SiaT: 9.4 × 10^2^ U/g cell dry mass; CSS: 3.4 × 10^2^ U/g cell dry mass). Using this catalyst, we show 3SL production at 40 ± 4 g/L in ∼ 70% yield within 3 h, reaching a cell dry mass-specific productivity of ∼ 3 g/(g h) and avoiding intermediary CMP-Neu5Ac accumulation almost completely (≤ 10% of total reactant mass). Overall, this study demonstrates an integrative engineering approach towards a high-flux sialylation module in *E. coli*. This module can promote flexible bioengineering of whole-cell catalysis for sialo-oligosaccharide production.

## Results and discussion

### Host strain and co-expression system used

The substrates of 3SL synthesis (Fig. 1) can be degraded by enzymes of the native *E. coli* background. Several studies [22, 24, 49] have therefore used individual or combined knock-out of LacZ β-galactosidase and Neu5Ac lyase (NAL). We show with direct activity measurements in the induced *E. coli* BL21(DE3) cell extract that NAL is below detection (Additional file 1: Fig. S1) and that β-galactosidase is present at ~ 5 U/g_cdm_. The β-galactosidase activity was recorded with ortho-nitrophenyl-β-galactoside which is a 12.5-fold better substrate of the enzyme than lactose [50]. CSS and α2,3-SiaT are well expressed as single enzymes in *E. coli* [41]. With the plausible assumption, based on the earlier work on single enzyme expression [41], that each co-expressed enzyme will account for at least ~ 10% of total intracellular protein in the recombinant *E. coli*, we expected whole cell activities of CSS and α2,3-SiaT in the approximate range 3 × 10^2^–2 × 10^3^ U/g_cdm_ (see also later in “Results and discussion” section). These activities exceed the β-galactosidase background on lactose by ≥ 750-fold. The native *E. coli* strain without additional knock-outs therefore appeared to be suitable for whole cell catalyst development. The β-galactosidase knock-out strain was therefore not pursued here.

Based on our previous research [42], we constructed four plasmids for CSS and α2,3-SiaT co-expression. As shown in Additional file 1: Tables S4, S5, Fig. S2, two bicistronic (i.e., two consecutive open reading frames controlled by one promotor) plasmids referred to as pBICI involve expression control by P_T7_ and have the α2,3-SiaT gene placed before the CSS gene. They differ in the use of strong (pBICI_strong) and weak (pBICI_weak) ribosome binding sites (RBS), as described in our earlier work [42]. Two monocistronic plasmids (i.e., two independent open reading frames controlled by individual promotors) referred to as pDUAL involve the α2,3-SiaT gene under control of P_T7_ and the CSS gene under variable control by promoter (P_T5_, P_*tac*I_) and RBS (Additional file 1: Fig. S2). Three plasmids (pBICI_weak, pDUAL_T5, pDUAL_tacI) were promising to achieve balanced enzyme co-expression. By contrast, pBICI_strong was expected to yield CSS activity in excess.

### Co-expression strategy guided by kinetic modeling

**Fig. 2:**
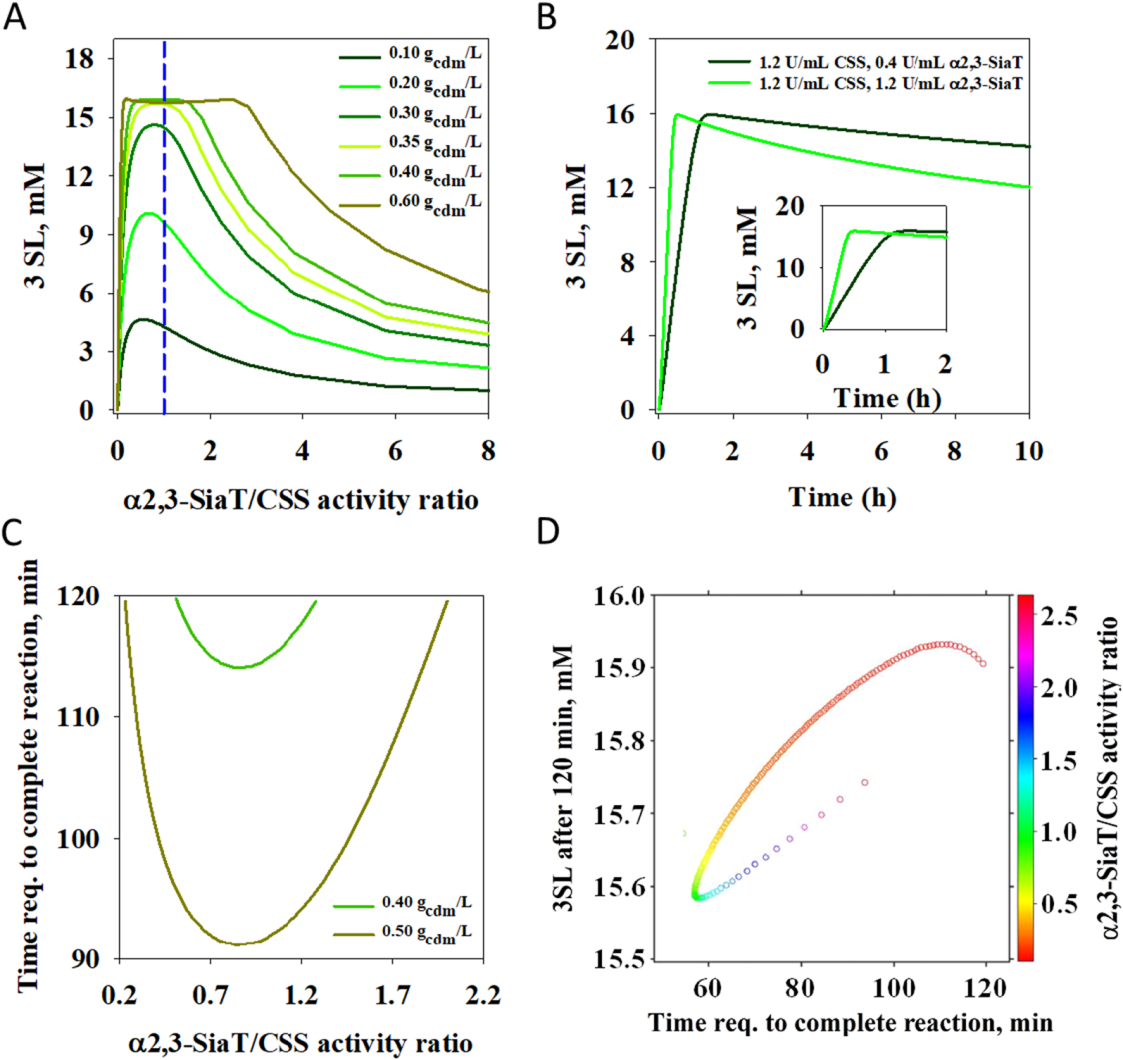
Kinetic simulations of whole cell-catalyzed production of 3SL. The 3SL release in reactions of 20 mM substrate (CTP, Neu5Ac, lactose) is shown for an assumed *E. coli* cell that co-expresses 150 mg total recombinant protein/g_cdm_ in variable relative composition of α2,3-SiaT and CSS. The specific activity of the enzymes (CSS: 36 U/mg; α2,3-SiaT: 5.7 U/mg) leads to the activity ratios shown in **A**. Reactions for 2 h at variable cell loadings. The dashed vertical line shows results for a perfectly balanced enzyme ratio. **B** Time courses of 3SL release at varied activity ratio. **C** Analysis of minimum time required for maximum 3SL release (16 mM). **D** Effect of enzyme ratio on time to maximum 3SL and on 3SL remaining after 2 h, determined for a catalyst loading of 0.80 g_cdm_/L

Kinetic model of the coupled CSS and α2,3-SiaT reaction (Additional file 1: Equations S1–S8, Table S3) was used to simulate the substrate conversion and the product release dependent on key variables of whole cell-catalyzed transformation. In particular, we analyzed effect of the enzyme ratio α2,3-SiaT/CSS, assuming that the cell produces 150 mg of total enzyme/g_cdm_. The assumption of ~ 15% share of target enzyme in the total cell mass appeared realistic, which is also confirmed later when showing the experimental results of enzyme co-expression (see Table 1). However, it should be noted that the assumption used at this stage did not in any way prejudice the course of the research and the results obtained in it. It is simply a parameter that needed to be fixed for the purpose of simulation. The simulations also analyzed the effect of loading of cell catalyst. The effect turns out to be non-trivial in being strongly interconnected with the effect of the enzyme ratio. Figure 2 shows simulation results for 3SL release from 20 mM substrate solution (Neu5Ac, lactose, CTP) at a fixed reaction time of 2 h. Under the conditions used, the combined effect of partial hydrolysis of CMP-Neu5Ac and equilibrium position of the α2,3-SiaT reaction limits the 3SL formation to ~ 80% (16 mM) of the CTP used, as also shown in earlier studies conducted with isolated enzymes [41]. The results (Fig. 2A) show that a minimum loading of cell catalyst (~ 0.40 g_cdm_/L) is required to reach the maximum of 3SL release.

At each cell loading, as revealed in Fig. 2A, dependence of the 3SL concentration on the enzyme activity ratio α2,3-SiaT/CSS passes through a distinct maximum and decreases sharply at higher values of the activity ratio. The position of the maximum shifts to higher activity ratios and becomes broader as the catalyst loading is increased. The 3SL maximum at low cell loading (≤ 0.20 g_cdm_/L) is found at an activity ratio of α2,3-SiaT and CSS distinctly smaller than unity (≤ 0.6). The effect is explained by the requirement of a basal level of CMP-Neu5Ac formed in the reaction by CSS in order to utilize the activity of α2,3-SiaT efficiently. The α2,3-SiaT exhibits a relatively high *K*_m_ of 1.0 mM for CMP-Neu5Ac (Additional file 1: Table S3). Interestingly, at higher catalyst loadings (> 0.60 g_cdm_/L), a decrease in the maximum concentration of 3′-sialyllactose (3SL) is observed for certain enzyme combinations. This is attributed to the reverse reaction of α2,3-SiaT (3SL + CMP → CMP-Neu5Ac + lactose) which releases CMP-Neu5Ac that can be hydrolyzed by the enzyme (CMP-Neu5Ac → CMP + Neu5Ac). Removal of CMP-Neu5Ac from the equilibrium of the transferase reaction leads to a slow degradation of 3SL once CTP is completely consumed and no new CMP-Neu5Ac can be formed (Fig. 2B).

Figure 2C compares different enzyme ratios enabling maximum 3SL formation within the 2 h window, in terms of the *actual* time required for this process to occur. The simulations done at catalyst loadings of 0.40 and 0.50 g_cdm_/L reveal the corresponding optimum enzyme ratio for minimum reaction time needed. The optimum ratio is similar, but not identical for the different catalyst loadings. Effect of 3SL degradation is not significant within 2 h in the simulations shown in Fig. 2C, yet it becomes relevant at higher loadings of catalyst. Figure 2D reveals the requirement for kinetic control of synthetic reaction for an assumed catalyst loading of 0.80 g_cdm_/L. Within the optimum range of enzyme activity ratio centered at ∼1, the time needed for maximum 3SL release is just below 60 min. Allowing the reaction to proceed for 2 h would already result in a slightly decreased product yield.

In summary, the results in Fig. 2 establish a basic framework for the design of whole cell catalysts in terms of α2,3-SiaT/CSS activity ratio and the use of these catalysts in the reaction. Figure 2 also suggests that cell loading can, to a certain degree, compensate an activity ratio that is not optimally balanced. Based on a cell loading of 0.40 g_cdm_/L, the suitable enzyme composition would range from 12 to 22% CSS of total co-expressed protein. Generally, the suitable range of enzyme composition gets narrower as the cell concentration used is lowered. A further point deduced from the simulation results is that full conversion in 2 h requires a minimum catalyst concentration of 6.6 mg/mL CSS and 50 mg/mL α2,3-SiaT. These results are in good agreement with earlier results of sialylation cascade characterization and optimization by the soluble enzymes [41].

### Co-expression results

**Fig. 3:**
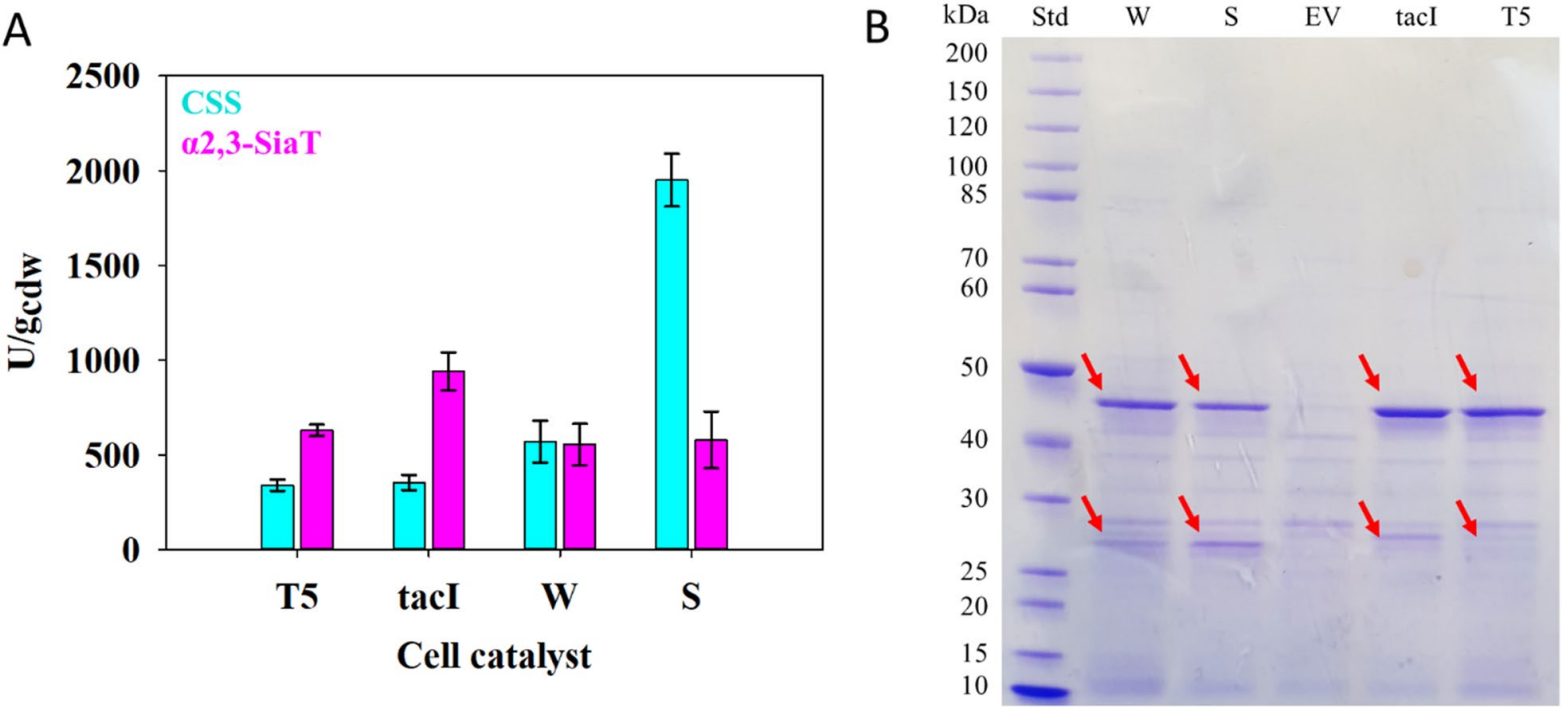
Co-expression results for CSS and α2,3-SiaT analyzed by enzyme activity (**A**) and SDS PAGE (**B**). The expression was here done at 25 °C. HPLC assay was used for activity determination (see “Methods”) at 37 °C. The SDS polyacrylamide gel shows the soluble fraction received by *E. coli* cell disruption. CSS (26.1 kDa) and α2,3-SiaT (47.2 kDa) are highlighted with arrows. Assignment of protein band to enzyme was based on molecular mass. Activity measurements shown in panel A confirm the presence of the enzyme. Molecular mass standard (Std); pBICI_weak (W); pBICI_strong (S); pDUAL_tacI (tacI); pDUAL_T5 (T5), empty vector (EV)

The four co-expression constructs were analyzed and the results are shown in Fig. 3. Table 1 gives a summary of the enzyme co-expression whereby the protein data are calculated from the measured activities with the known specific activities of CSS and α2,3-SiaT. Activity comparisons (Fig. 3A) reveal that the pBICI_weak construct achieves the goal of balancing the difference in CSS and α2,3-SiaT specific activity via differential protein co-expression (activity ratio: ~ 1). The activity ratio is squarely within the window of optimum co-expression for a complete conversion (~ 14% CSS of total protein co-expression) at a cell loading of 0.40 g_cdm_/L. The pDUAL constructs even establish a moderate excess of the α2,3-SiaT activity. The relative content of CSS is ~ 8% (pDUAL_T5) and ~ 7% (pDUAL_tacI). For a cell loading of 0.40 g_cdm_/L, therefore, these cell catalysts might not be able to reach full conversion in the conditions of Fig. 2. The pBICI_strong construct achieves a ~ threefold excess of CSS activity (Fig. 3A; Table 1). With this catalyst applied at 0.40 g_cdm_/L, the α2,3-SiaT activity may thus become borderline for full conversion. As recognized from Fig. 3A, the pBICI_strong construct involves the interesting feature that the less favorable enzyme activity ratio results from a very high CSS activity that is formed next to a α2,3-SiaT activity comparable to that received with other constructs (pDUAL_T5; pBICI_weak). Comparing pBICI_strong to pBICI_weak, we find that both constructs give a similar expression level of α2,3-SiaT (~ 570 U/g_cdm_). The expression of CSS is upregulated in pBICI_strong due to the relatively stronger RBS, giving 1950 U/g_cdm_ compared to 570 U/g_cdm_ in pBICI_weak. Analysis by SDS PAGE reveals differential protein co-expression dependent on the plasmid used (Fig. 3B) and the evidence supports the results of the activity measurement. As shown in Table 1, the cell catalyst differs in total protein expression by about 1.6-fold. The protein ratio of CSS and α2,3-SiaT changes by ~ tenfold from 1:19 in pDUAL_tacI to 1:1.9 in pBICI_strong (Table 1).

**Table 1:** Parameters of whole cell catalyst and reaction for synthesis of 3SL

	Activity (protein) ratio CSS:α2,3-SiaT	catalyst concentration [g_cdm_/L]^a^	3SL [g/L]	Specific production rate [g 3SL/g_cdm_/h]^c^	*STY* [g/(L h)]^d^	*TTN* [–]^e^
pBICI_weak (113 mg/g_cdm_)	1:1 (1:6.3)	0.58	8.2 (69)^b^	16	6.2	14
pBICI_strong (145 mg/g_cdm_)	1:0.3 (1:1.9)	0.22	5.1 (42)	23	4.0	23
pDUAL_tacI (175 mg/g_cdm_)	1:3 (1:19)	0.94	8.5 (68)	13	8.1	9.0
pDUAL_T5 (119 mg/g_cdm_)	1:2 (1:13)	0.98	8.3 (70)	11	7.5	8.5

^a^Cell concentration used for CSS activity of 0.4 U/mL; 37 °C, 20 mM substrate

^b^3SL yield (based on 20 mM substrate used) shown in brackets

^c^Calculated for the 30 min reaction

^d^Calculated for the 1 h reaction

^e^*TTN* = g_3SL_ after 2 h/g_cdm_

In a comparison of the different constructs, we must also consider that the limiting CSS activity is ~ twofold higher in pBICI_weak (570 U/g_cdm_) compared to pDUAL_tacI (353 U/g_cdm_) and pDUAL_T5 (340 U/g_cdm_) (Fig. 3A). The result implies that pBICI_weak will require a lower catalyst loading than pDUAL_tacI and pDUAL_T5 in order to accomplish the same conversion task. Here, all four cell catalysts were assessed systematically for performance in 3SL synthesis.

### Whole cell catalyst evaluation for 3SL synthesis

**Fig. 4:**
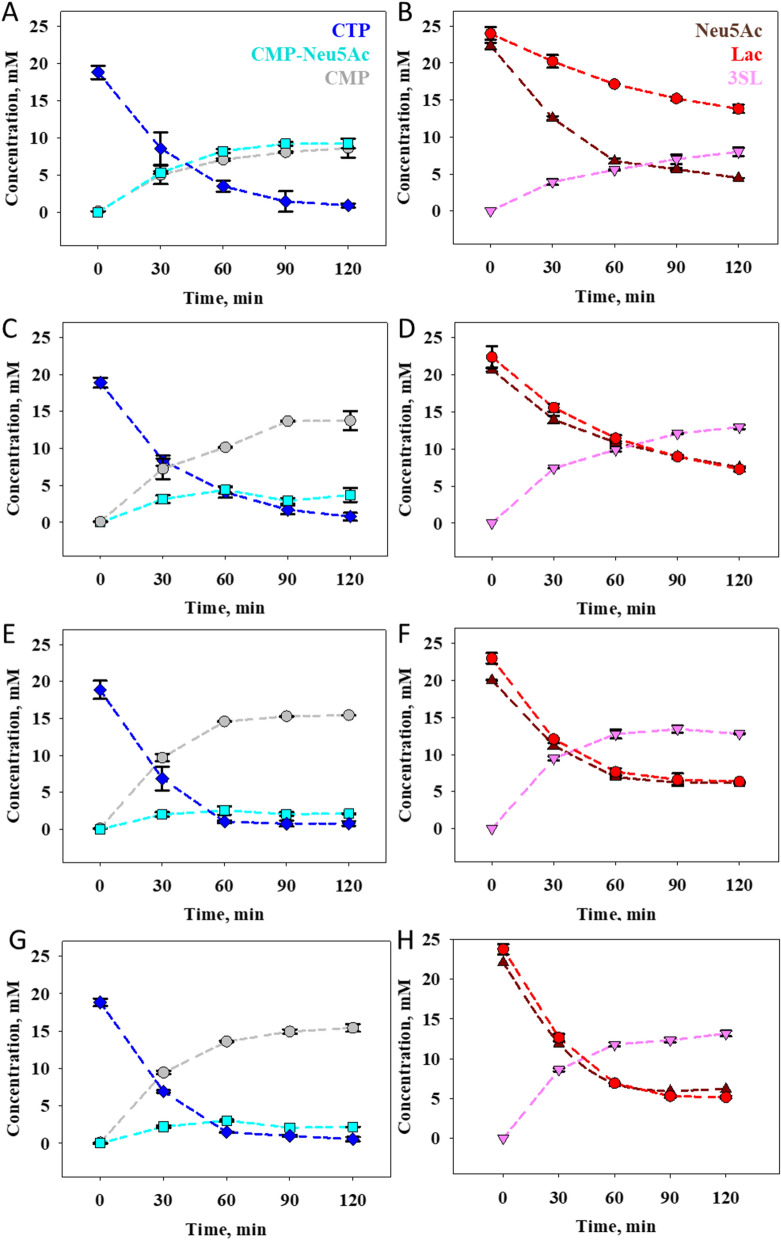
Reaction time courses of whole cell-catalyzed 3SL synthesis. The reactions are performed with ~ 20 mM of each Neu5Ac, CTP and lactose at 37 °C and pH 8.0. The CSS activity is normalized to 0.40 U/mL in each reaction based on variable loading of the cell catalyst, hence also activity ratio (*R*_E_) of CSS:α2,3-SiaT, as follows. **A**, **B** 0.22 g_cdm_/L, pBICI_strong, *R*_E_ 1:0.3; **C**, **D** 0.58 g_cdm_/L, pBICI_weak, *R*_E_ 1:1; **E**, **F** 0.94 g_cdm_/L, pDUAL_tacI, *R*_E_ 1:3; **G**, **H** 0.98 g_cdm_/L, pDUAL_T5, *R*_E_ 1:2). Symbols show the mean values of 3 replicate experiments and error bars show the S.D

To analyze the reactions of the different cell catalysts, we selected a constant CSS activity of 0.40 U/mL, adjusted by a variable concentration of cell catalyst used in the range 0.22–0.98 g_cdm_/L (Table 1). The CSS activity was set to allow for full conversion of 20 mM substrate in an estimated time of ~ 100 min, assuming a net rate over the two enzymatic steps that is minimally half the CSS rate, therefore: 20 [µmol/mL] × 1/0.2 [mL/(µmol min)] = 100 min. We wish to clarify that CTP was used as substrate in the equivalent concentration of Neu5Ac and lactose. The conditions are meant for characterization and optimization of the co-expression for whole cell catalyst development. They are not meant for production because production could only be economical with CTP regeneration. Comprehensive time course data for the different reactions are shown in Fig. 4. The possibility of 3SL degradation at extended reaction times when high cell loadings are used (see Fig. 2B, D) underlines the importance of time course analysis. The results shown here are validated by close mass balance of the reaction. The total Neu5Ac found in 3SL, CMP-Neu5Ac and free Neu5Ac at each point of the reaction agreed very well (≤ 5%) with the initially added Neu5Ac. These results effectively exclude the possibility of side reactions occurring in a substantial amount. They are important in light of a recent study by Konietzny et al. [51] who discovered that the sialyltransferase PmST1 from *Pasteurella multocida* catalyzes sialyltransfer from CMP-Neu5Ac to tris(hydroxymethyl)aminomethane (Tris). Note that Tris is the buffer used in our experiments. PmST1 is furthermore related to the α2,3-SiaT used here by common membership to family GT80 of the glycosyltransferase families. Caution is therefore required in the analysis of sialyltransferase reactions performed in Tris buffer and close mass balance must be ensured (see above).

Evidence that time courses of CTP utilization are almost superimposable in all reactions verifies the intended use of an identical CSS activity in these experiments. Figure 4A shows that the pBICI_strong catalyst gives accumulation of CMP-Neu5Ac in amounts comparable to 3SL released. There is accordingly a large gap between Neu5Ac and lactose consumed (Fig. 4B). The pBICI_weak (Fig. 4C, D) and pDUAL_tacI (Fig. 4E, F) catalysts give lowered accumulation of CMP-Neu5Ac in that order. The pDUAL_T5 catalyst (Fig. 4G, H) gives effectively the same conversion profile as the pDUAL_tacI catalyst. The 3SL release is consistently lower by ~ 15% in all reactions than the CMP release, which results due to the hydrolysis of CMP-Neu5Ac by the α2,3-SiaT (see Additional file 1: Fig. S3). Evidently, the occurrence of CMP-Neu5Ac hydrolysis renders CTP the substrate that is limiting for the maximum amount of 3SL released in the overall reaction.

Table 1 summarizes key engineering parameters of the whole cell-catalyzed transformations determined from the data in Fig. 4. Based on 3SL release in the first 5–30 min, the specific production rate (g 3SL/g_cdm_/h) is highest for pBICI_strong, exceeding that of the other catalysts by up to 2.1-fold. The pDUAL_tacI and pDUAL_T5 catalysts show the lowest specific rate. Reactions of the catalysts pBICI_weak, pDUAL_tacI and pDUAL_T5 release 3SL to ∼14 mM, corresponding to ~ 70% conversion of the CTP used. The pBICI_strong catalyst gives only ∼ 42% conversion, consistent with the model predictions from Fig. 2: the concentration of pBICI_strong cell catalyst is just too low for maximum conversion in 2 h. The space-time-yield (*STY*) calculated after 1 h places the pDUAL_tacI and pDUAL_T5 reactions before the pBICI_weak reaction. The pBICI_strong reaction shows the lowest *STY*. In terms of mass-based turnover number of catalyst (*TTN*), the pBICI_strong construct surpasses the other constructs by up to ∼threefold. For high-yield synthesis of 3SL, however, shortcoming of the pBICI_strong construct is too low α2,3-SiaT activity.

As pointed out in “Co-expression strategy guided by kinetic modeling” section while discussing the simulation results, there is a minimum concentration of 50 mg/L α2,3-SiaT required for maximum 3SL release in 2 h. The α2,3-SiaT concentration used in the reaction of Fig. 4A, B is just 21 mg/L. In other words, to reach the target conversion, the loading of pBICI_strong catalyst would have to be increased from the 0.22 g_cdm_/L used in the constant CSS conditions (Fig. 4) to 0.52 g_cdm_/L. At this catalyst loading, it may be possible that the pBICI_strong reaction avoids the substantial accumulation of CMP-Neu5Ac after 2 h, seen in Fig. 4A.

**Fig. 5:**
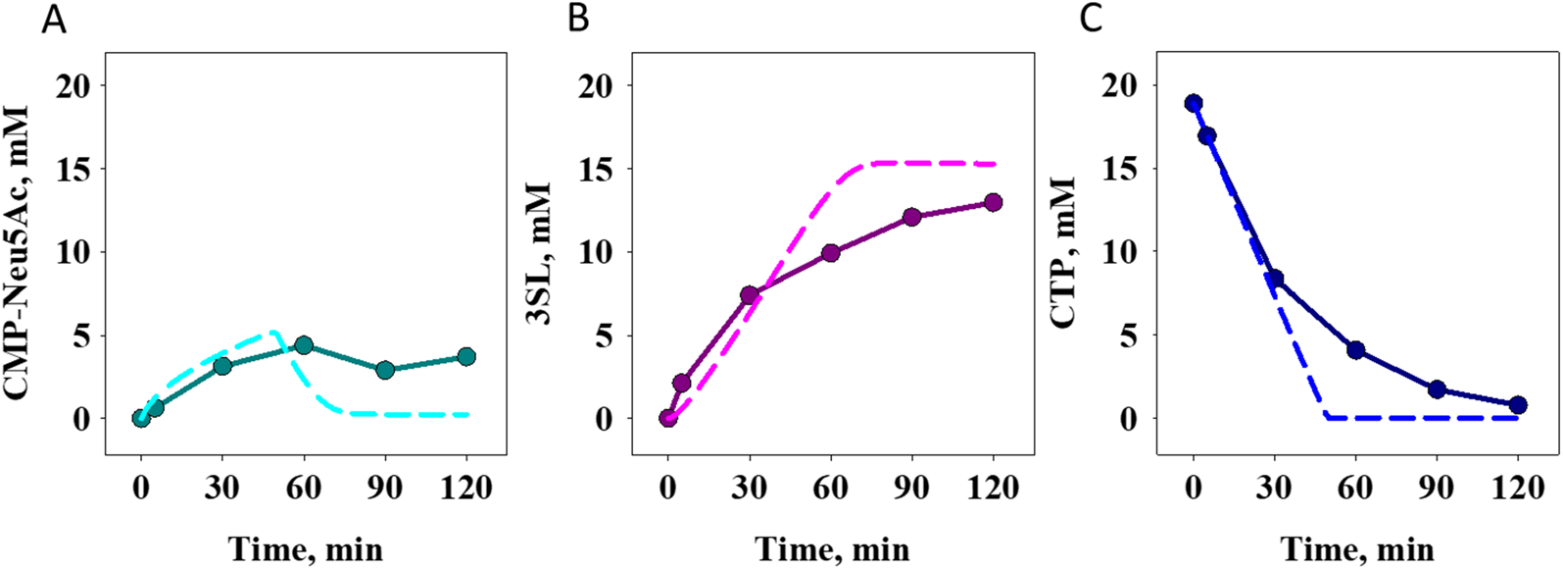
Simulated time courses of the pBICI_weak reaction compared to experimental data. The data (**A** CMP-Neu5Ac; **B** 3SL; **C** CTP) are from Fig. 4C, D. Simulation is shown with dashed line

To further analyze the conversion experiments, all time courses of Fig. 4 were simulated with the kinetic model. Simulations show overall good agreement with experiment in the initial reaction phase, while divergence between the two occurs later in the transformation (data not shown). The 3SL release slows down at a concentration much lower than expected from the model. The effect can be observed in all experiments, which theoretically should achieve full conversion (Table 1) and is discussed here using the catalyst pBICI_weak as an example (Fig. 5). The model predicts that the intermediate CMP-Neu5Ac passes through a maximum and then decreases as the reaction proceeds (Fig. 5A). In the experiment, CMP-Neu5Ac actually increases beyond the expected maximum and does not decrease toward the end of the reaction, resulting in a large gap between modeled and measured 3SL (Fig. 5B). The time course of CTP consumption shows a complete consumption well in agreement between experiment and model after 2 h, however here the consumption of CTP also slows down similarly to the synthesis of 3SL (Fig. 5C).

In conclusion, based solely on the experimental conversion results in Fig. 4, pBICI_weak appears to be the best candidate construct out of the four constructs evaluated in order to achieve a balanced 3SL synthesis at minimal loading of cell catalyst. While both pDUAL constructs also fulfil the minimum requirements for full conversion under the conditions used, the pBICI_weak construct offers the advantage of a ~ 2-times higher CSS activity. Additionally, pBICI_weak maintains a balanced enzymatic activity ratio, different from pBICI_strong. However, as shown in Fig. 5B, the 3SL released in the experiment is lower by ∼ 15% than predicted by the model, suggesting factor(s) not yet identified controlling the conversion at longer reaction times. Enzyme stability was therefore analyzed.

### Enzyme stability as a “hidden” factor of 3SL production efficiency

**Fig. 6:**
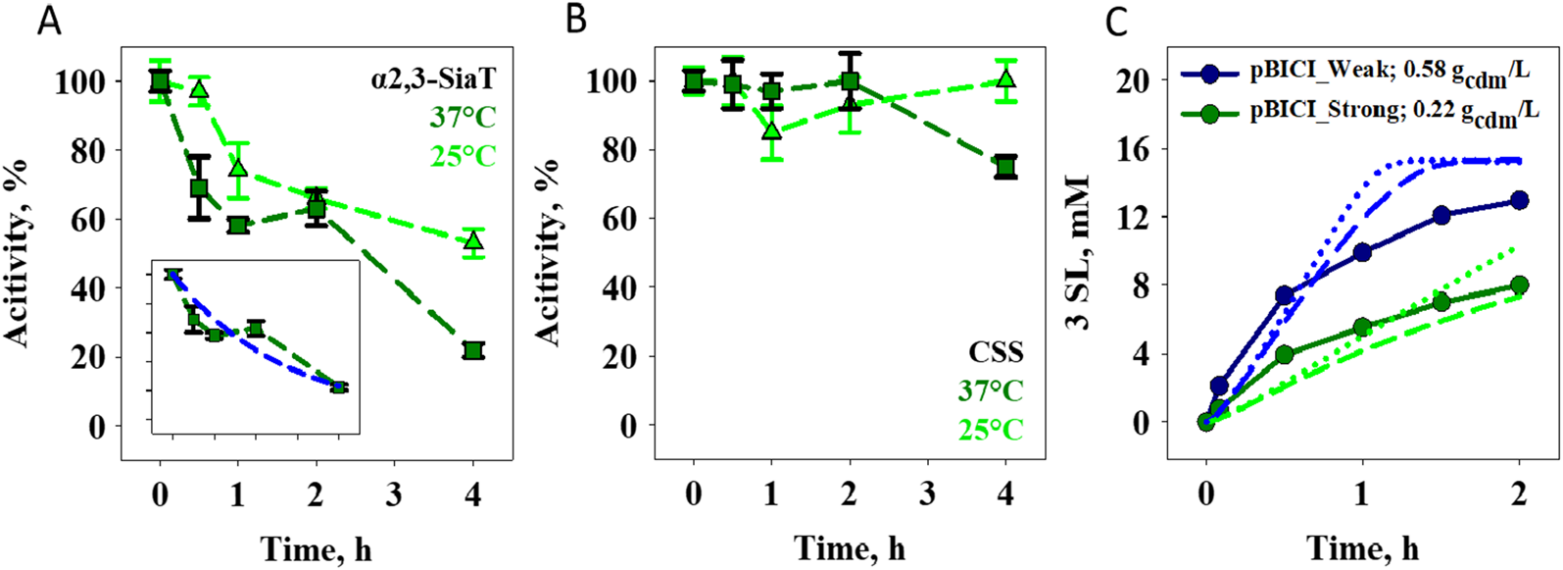
Enzyme stability in whole cell catalysts. **A** α2,3-SiaT and **B** CSS in pBICI_strong. The catalyst was incubated (pH 8.0, 450 rpm) at 37 °C (squares) or 25 °C (triangles). Activities (photometric assay) are mean values of three replicate experiments and error bars show the S.D. Symbols show the data and dashed lines indicate the trend. The inset of panel **A** shows exponential fit (dashed blue line) of the data. **C** Simulation of 3SL release in the absence of (dotted lines) and under the assumed involvement of inactivation of α2,3-SiaT (dashed lines), compared to experimental data at 37 °C. A first-order inactivation constant of 6.1 × 10^−3^ min^−1^ (equivalent to a half-life of 110 min) was assumed

The individual stability of CSS (Fig. 6A) and α2,3-SiaT (Fig. 6B) was assessed in cell catalyst preparation incubated at 37 °C as in the reactions. α2,3-SiaT appears to inactivate faster considerably than CSS. The α2,3-SiaT half-life is estimated as just ~ 110 min. The inactivation of α2,3-SiaT is slowed down by temperature decrease to 25 °C, roughly by a factor of 2 (Fig. 6A). However, whereas these results point to a limited robustness of the α2,3-SiaT, they do not explain the fast decline of reaction progress in terms of 3SL release and CTP consumption, as shown in Fig. 5B, C. Additionally, they cannot account for the dynamics of the CMP-Neu5Ac intermediate (Fig. 5A). The absence of decrease in the CMP-Neu5Ac concentration as observed experimentally (Fig. 5A) seems to indicate loss of α2,3-SiaT activity in substantial extent already at 60 min and even earlier. Simulations (Fig. 6C) show furthermore that the characteristics of reaction time courses by the different cell catalysts cannot be described with a single inactivation rate constant for α2,3-SiaT. While the current evidence supports the idea of reaction slowdown caused by α2,3-SiaT activity loss, the underlying mechanism is not resolved. As a similarly fast loss of α2,3-SiaT activity was not detected in studies of the isolated enzymes [41], it is tempting to speculate that the whole cells and the microenvironment in them are relevant factors. Tentatively, microscale pH gradient between the cell and the bulk liquid might be important (see [52] for pH change associated with the reactions). Further studies, however beyond the scope of the current inquiry, will be needed for clarification.

We analyzed 3SL synthesis at the lowered temperature of 25 °C. The pBICI_strong catalyst was used. The idea was that given the low abundance of α2,3-SiaT in this catalyst (Table 1), the stabilization of α2,3-SiaT should have a pronounced effect on the reaction time course that one should be able to conveniently detect in the experiment. However, while performing the comparative study of the pBICI_strong reaction at 25 and 37 °C, we noted that the CSS activity is affected more strongly by the temperature change than the α2,3-SiaT activity. The CSS:α2,3-SiaT activity thus changes from 1:0.5 at 37 °C to 1:3 at 25 °C. Comparison of the two reactions at a temperature-independent CSS activity of 0.4 U/mL reveals the interesting effect that reaction at 25 °C involves a more complete release of 3SL due to a lower amount of CMP-Neu5Ac accumulating (Additional file 1: Fig. S4).

### Reaction intensification for whole cell-catalyzed 3SL production

**Fig. 7:**
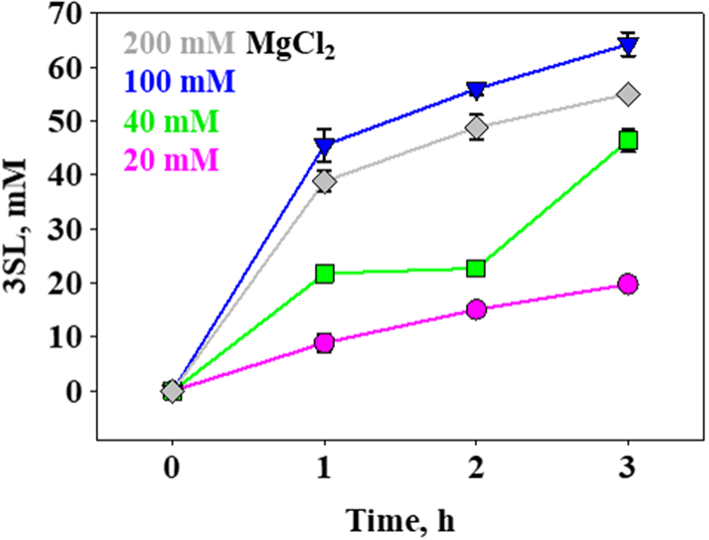
Intensification of 3SL synthesis by pDUAL_tacI catalyst. Reactions were performed at 25 °C using 100 mM of each Neu5Ac, CTP and lactose. The MgCl_2_ concentration (mM) was varied as indicated in the figure. The 3SL was analyzed by HPLC. Symbols show the data which are averages from 3 replicate experiments. Error bars show the S.D

Insight about the role of α2,3-SiaT stability in determining the overall reaction efficiency required us to reconsider the choice of cell catalyst best suited for 3SL synthesis. Although pDUAL_tacI exhibits a lower CSS activity than pBICI_weak, its excess of α2,3-SiaT activity over pBICI_weak (Table 1) appeared as a decisive advantage for whole cell-catalyzed reaction under conditions of elevated substrate concentration (here: 100 mM). Decrease in temperature to 25 °C results in a change in the CSS:α2,3-SiaT activity ratio from 1:3 at 37 °C (Table 1) to now 1:20 and so it further increases the α2,3-SiaT activity available in excess. The cell loading was adjusted (13 g_cdm_/L) based on the limiting CSS activity (2.0 U/mL at 25 °C), to potentially allow for complete substrate conversion into 3SL (∼ 80% of CTP used, as suggested by the model) within 2 h. Considering the expected requirement of increased Mg^2+^ due to the higher concentration of CTP used, experiments were performed at a varied concentration of Mg^2+^ in the range 40–200 mM. As shown in Fig. 7, reaction at 100 mM Mg^2+^ gives the best result of 67 mM 3SL released after 3 h. The conversion under these conditions proceeds ∼ twofold slower than predicted by the kinetic model, used with the assumption that *K*_m_ values (Additional file 1: Table S3) are unchanged by the temperature decrease to 25 °C. Loading of cell catalyst that was ∼13-fold enhanced compared to the reaction at 20 mM substrate (Table 1) might be a relevant factor. Further research is warranted to achieve clarification. Parameters of reaction efficiency determined from Fig. 7 are the following: nominal 3SL yield of 67% (± 2%), corresponding to 85% of the maximum reaction output limited by hydrolysis; 3SL concentration of 40 (± 4) g/L; 3SL *STY* of 14 (± 2) g/L/h; and *TTN* of 3.0 (± 0.3) g 3SL/g_cdm_. These results are comparable to earlier reports, working with cell mixtures [26, 40], in terms of the 3SL produced (≤ 36 g/L). They however represent significant improvements in *STY* and *TTN* by ∼ fivefold and ≥ threefold, respectively. Overall, these findings show promise to combine the here developed sialylation module with a dedicated module of CTP regeneration for whole cell-catalyzed production of 3SL. As already mentioned, however, enzyme stability will require attention in further development.

### In silico prototyping of enzyme cascade for efficient whole cell catalysis

Given the high interest in multi-enzymatic cascades applied to the production of pharmaceuticals [53] and more recently also commodity chemicals [54, 55], efficient strategies for the development of the required biocatalysts become increasingly important. As several studies have pointed out (e.g. [15, 56–60]), the optimization of cascade reactions involves the challenging task of catalyst design according to the complex max-min criterion that the composition of individual enzymes should enable maximum overall flux through the multi-step cascade at minimum total mass of catalytic protein used. Based on earlier work done with cell-free enzyme preparations [41], the approach used here was systematic kinetic model-based prototyping of the enzyme composition in order to inform differential protein co-expression in *E. coli* for experimental development of a whole cell catalyst consistent with the stated max–min principle. Using term from metabolic engineering, a detailed model-based flux control analysis [61–63] is performed for the bi-enzymatic cascade of 3SL synthesis. As far as we know, the approach is novel in the broader field of applied biocatalysis and is expected to have relevance in general at the point when upscaling of multi-enzymatic cascade transformation is considered. At this particular point of development, the question of the catalyst preparation used necessitates a definite answer; and a cell catalyst that co-expresses the required set of enzymes in a ratio suitable for efficient reaction presents an attractive solution. The cell catalyst overcomes the drawback of having to produce each enzyme individually. The current study supports the important use of kinetic modeling to guide enzyme co-expression in an integrative development that considers the whole cell catalyst as well as the biotransformation for production.

Lastly, we consider the possible reservation against co-expression optimization of a small two-enzyme module when the actual biocatalytic transformation requires the additional enzymes of the CTP module. In a reaction for economical synthesis, CTP cannot be used as substrate and must be generated in situ. The use of CTP substrate in our study was for development, not for production. However, the multienzymatic modules for sialylation and CTP regeneration can be developed individually in separate *E. coli* whole cell catalysts. These catalysts can then be combined in a one-pot reaction as shown in earlier works of SL production [26, 40]. The exchange of reactants between the whole cell catalysts is enabled by cell permeabilization. While optimal interfacing of the modules for sialylation and CTP regeneration might still involve further fine tuning of α2,3-SiaT and CSS, there is no reason to believe that the basically optimized module for sialylation could not be established independently, as shown in this work. If instead of separate expression of the two modules their co-expression in a single strain of *E. coli* was preferred, one should certainly be prepared to encounter mutual interference from the different enzymes produced recombinantly. However, any approach of module co-expression would benefit from the thorough individual characterization of the modules used.

## Conclusions

Co-expression modules for α2,3-sialyltransferase and CMP-sialic acid synthetase in *E. coli* were established. Imbalance in the specific activities of the two enzymes was overcome by coordinated co-expression using different designs of plasmid vector. Kinetic model-based in silico prototyping was used for optimization of the enzyme ratio in whole cells thus enabled to efficient conversion of substrates (Neu5Ac, CTP, lactose) into 3SL. Permeabilized cells show high α2,3-SiaT and CSS activity of 9.4 × 10^2^ U/g_cdm_ 3.4 × 10^2^ U/g_cdm_, respectively. Sialyltransferase stability was identified as an important factor of conversion efficiency. The balanced co-expression of CSS and SiaT yields an efficient (high-flux) sialylation module for flexible development of *E. coli* (e.g., food-grade strain *E. coli* Nissle 1917, [64]) as whole-cell catalysts for sialo-oligosaccharide production.

## Methods

### Materials, genes, plasmids and microorganism

CTP (95% purity; 5% CDP), CMP, CMP-Neu5Ac, 3SL (all sodium salt), Neu5Ac and *N*-acetyl-d-galactosamine (GalNAc) were from Carbosynth (Compton, Berkshire, UK). Other chemicals were of reagent grade. The *N. meningitidis* CSS (GenBank: U60146.1; Uniprot: P0A0Z8) and the *P. dagmatis* α2,3-SiaT (GenBank: JX870648.1; Uniprot: K9UUI6) were used. The α2,3-SiaT construct involves the three-residue (Lys-Thr-Ile-) N-terminal extension for enhanced functional expression in *E. coli* [65]. Both enzymes are equipped with a non-cleavable hexahistidine tag placed N-terminally in CSS and C-terminally in α2,3-SiaT and are the same as previously used for development of the kinetic model [41]. The specific activities of α2,3-SiaT (5.7 U/mg) and CSS (36 U/mg) reported in Schelch et al. [41] for the enzymes purified from single expression *E. coli* culture were re-confirmed in this work. Mono- and bicistronic plasmid vectors, reported previously [42] and referred to as pDUAL and pBICI, respectively, were used for enzyme co-expression. Cloning of CSS and α2,3-SiaT into the co-expression vectors is described in detail in Additional file 1. *E. coli* BL21(DE3) was used as host. A full list of the strains, genes and plasmids used is given in Additional file 1: Table S1.

### Whole cell catalyst

#### Preparation

Strains were grown in LB media (100 mg/L ampicillin) at 37 °C using 50 mL volume in 300-mL baffled shake flasks at 110 rpm agitation rate (CERTOMAT BS-1; Sartorius). Induced cultures (0.4 mM isopropyl β-d-1-thiogalactopyranoside; added at an optical density of ~ 1.0) were further incubated at 18 or 25 °C with time of harvest varied at 2, 5 or 21 h. Centrifuged cells (20 min, 4 °C, 5000 rpm, Ultracentrifuge Sorvall RC-5B Superspeed) were re-suspended in 0.9% NaCl at ~ 100 mg wet cell mass/mL and frozen in 0.5 mL aliquots at − 20 °C for ≥ 14 h until further use (no loss of both enzyme activities was observed for at least 2 weeks). We refer to cells having undergone freeze-thaw as permeabilized. The procedure was shown to be highly reproducible (S.D. ≤ 20%; *N* = 3) in terms of whole cell enzyme activity obtained. The freeze–thaw permeabilization is a mild procedure and shows useful effectiveness. Other protocols of permeabilization were not pursued.

#### Cell dry mass

Sartorius Humidity Tester MA 50 was used. Water content of wet cell mass was determined as 80%. Cell dry mass (cdm) is calculated from measurement of wet mass.

#### Cell extract

Cell suspension (~ 0.6 mL; 100 mg/mL) was transferred into 2 mL tubes containing 400 mg glass mill beads (diameter 0.5 mm; BioSpec Products Inc.). Cell disruption involved 5 cycles of manual vortexing (30 s; reax control with an attachment for 10 reaction tubes, Heidolph Instruments) followed by 1 min storage on ice. Supernatant removed from the beads was centrifuged (13,200 rpm, 4 °C for 30 min, Centrifuge Eppendorf 5424 R) and both solid and soluble fractions analyzed for protein with Roti-Quant reagent (Roth) referenced to bovine serum albumin (BSA).

#### SDS PAGE

This was conducted using clarified cell extract (see above). The supernatant was adjusted to a protein concentration of ∼ 1 mg/mL, mixed with SDS loading buffer, and boiled for 10 min. Following this, 15 µL of the prepared mixture was loaded onto a 10% gel and electrophoresis performed at 200 V for about 60 min.

### Activity assays

#### pH shift method

pH shift assay from literature [52, 66] was adapted for fast semiquantitative measurement of CSS and α2,3-SiaT activity (2.0 mM Tris/HCl, pH 8.0). Both enzyme reactions involve release of proton that is detected by decrease in phenol red indicator (26 µM) absorbance at 560 nm. The CSS assay contained 2.0 mM Neu5Ac, 20 mM MgCl_2_, 0.2 mM l-Cys and 5.0 mM CTP; the α2,3-SiaT assay contained 1.0 mM CMP-Neu5Ac and 5.0 mM lactose. Assays were done in 96-well plates on a multi-mode microplate reader (FLUOstar® Omega, BMG LABTECH GmbH) at 25 or 37 °C. Four µL of diluted cell extract (final protein concentration 0.1–1 mg/mL) or permeabilized cells (final concentration 0.25–2.5 mg/mL) was added into a final volume of 150 µL. The microtiter plate was mixed for 0.5 min before absorbance decrease was monitored for 5–10 min. The assay was calibrated with known proton amounts based on HCl.

#### HPLC method

Activity assays were done in 200 µL total volume (0.1 M Tris/HCl; pH 8.0) using an Eppendorf Thermomixer comfort for temperature control (25 or 37 °C) and agitation (450 rpm). Permeabilized cells (final concentration 0.1–1.0 mg/mL) were added for assay start. The CSS reaction contained 20 mM CTP, 20 mM Neu5Ac, 40 mM MgCl_2_ and 0.2 mM L-Cys. Samples (20 µL) were mixed 1:1 with 1 M NaOH and analyzed by “nucleotide HPLC” (see “HPLC analysis” below). Activity refers to CMP-Neu5Ac formed. The α2,3-SiaT reaction contained 5.0 mM CMP-Neu5Ac, 20 mM lactose, 40 mM MgCl_2_ and 0.2 mM L-Cys. Samples (20 µl) heated (99 °C, 15 min) and analyzed with by “carbohydrate HPLC” (see “HPLC analysis” below). Activity refers to 3SL formed.

### HPLC analysis

#### Nucleotide HPLC

CTP, CDP, CMP and CMP-Neu5Ac were measured. Samples were mixed 1:1 with methanol (≥ 99.5%) and kept on ice for 20 min. Precipitate was centrifuged off (13,200 rpm; 4 °C, Centrifuge Eppendorf 5424 R, Eppendorf) and supernatant analyzed by ion-pairing HPLC on a Shimadzu SPD-20 A system with a Kinetex® 5 μm C-18 (100 Å; 50 × 4.6 mm) column. Tetra-*n*-butylammonium bromide (40 mM) in 20 mM potassium phosphate buffer (pH 5.9) was used. For elution, acetonitrile gradient (6.5–25%) was applied at a flowrate of 1.5 mL/min (40 °C). Detection was at 254 nm.

#### Carbohydrate HPLC

3SL, Neu5Ac, ManNAc and lactose were measured. Prior to analysis in order to avoid signal overlaps, the nucleotides present in the sample were degraded with alkaline phosphatase (Quick CIP, New England Biolabs, 41). The sample was spiked with GalNAc (final conc. 4.0 mM) as internal standard. Quick CIP (final concentration 50 U/mL; 1.5 µL) was mixed with sample (15 µL) into 20 mM Tris/HCl (pH 8.0) containing 40 mM MgCl_2_ to a final volume of 150 µL. The digest was performed at 30 °C and 450 rpm for 1 h. The sample was then heated to 70 °C (15 min) and centrifuged (13,200 rpm, 4 °C; Centrifuge Eppendorf 5424 R, Eppendorf). A LaChrom Merck Hitachi system with a BioRad Aminex® HPX-87 H column, a Merck Hitachi L-7400 UV detector (detection at 210 nm; 3SL, Neu5Ac, ManNAc) and a Merck LaChrom L-7490 RI detector (lactose) was used for analysis. The mobile phase was 5.0 mM H_2_SO_4_, with a flow rate of 0.5 mL/min at 65 °C.

### Kinetic modeling

Kinetic model of the coupled CSS and α2,3-SiaT reactions is adapted from [41] and shown in Additional file 1: Equations S1–S8. The model describes the forward reaction of the two enzymes, with approach to equilibrium for the α2,3-SiaT reaction accounted for by a mass action term. Apparent substrate binding (*K*_m_) is modeled as independent of the concentration of the respective other substrate. This simplifying assumption was validated in earlier work [41], showing that the model describes the dependence of the enzymatic rates on substrate concentration with sufficient accuracy. CMP-Neu5Ac hydrolysis by α2,3-SiaT is also implemented in the model. The model accounts for the change in CMP-Neu5Ac utilization via transfer to lactose and hydrolysis in dependence of the degree of saturation of α2,3-SiaT with the lactose acceptor. Reaction of permeabilized whole cells was modeled as unaffected by diffusion (Additional file 1: Table S2). Comparison to reaction of the corresponding cell extract (no change in rate compared to permeabilized cells) supports the assumption. The model involves the concentrations of CSS and α2,3-SiaT as experimental variables. In modeling whole cell reactions, the active protein was assessed from enzyme activity measurements of the whole cell preparation used, together with the known specific activities of isolated CSS (36 U/mg) and α2,3-SiaT (5.7 U/mg), as determined by Schelch et al. [41]. Simulations were performed in MATLAB (R2018). The model parameters used are summarized in Additional file 1: Table S3.

### Whole cell synthesis of 3SL

Standard reactions were done in 1.0 mL total volume using 0.1 M Tris/HCl buffer (pH 8.0) containing 40 mM MgCl_2_ and 0.2 mM l-Cys. Substrates (CTP, lactose, Neu5Ac) were used at 20 mM. Temperature (25 °C, 37 °C) and agitation (450 rpm) were controlled in a Thermomixer comfort instrument. Permeabilized cells were added in a concentration giving a CSS activity of 0.4 U/mL that was kept constant across experiments with different whole cell catalysts. Samples (20 µL) were taken up to 2 h and were analyzed by HPLC (see above) and TLC (Additional file 1).

Intensified reactions used substrates at 100 mM. The buffer was changed to 0.2 M Tris/HCl (pH 8.0; 0.2 mM l-Cys) and the MgCl_2_ was varied in the range 20–200 mM. The cell concentration was increased to a CSS activity equivalent of 2.0 U/mL.

### Supplementary Information

**Additional file 1:** Supporting information containing supporting methods, supporting tables, model equations and supporting figures.

## Data Availability

The data that support the findings of this study are available from the corresponding author upon reasonable request.

## Abbreviations

cdm: Cell dry mass
CMP: Cytidine 5′-monophosphate
CSS: CMP-sialic acid synthetase (EC 2.7.7.43)
CTP: Cytidine 5′-triphosphate
Neu5Ac: *N*-Acetyl-d-neuraminic acid
SiaT: α2,3-Sialyltransferase (EC 2.4.99.-)
3SL: 3′-Sialyllactose
