# Supplementary material for: Modular bioengineering of whole-cell catalysis for sialo-oligosaccharide production: coordinated co-expression of CMP-sialic acid synthetase and sialyltransferase
Source: Microb Cell Fact. 2023 Nov 27;22:241. doi: 10.1186/s12934-023-02249-1 (PMC10683312; doi:10.1186/s12934-023-02249-1)
Supplement: Supplementary file 1 — Additional file 1: Supporting information containing supporting methods, supporting tables, model equations and supporting figures. [file 12934_2023_2249_MOESM1_ESM.docx]

**ADDITIONAL FILE 1**

**Modular bioengineering of whole-cell catalysis for sialo-oligosaccharide production: coordinated co-expression of CMP-sialic acid synthase and sialyltransferase**

Sabine Schelch^a,b^, Manuel Eibinger^b^, Jasmin Zuson^a,b^, Jürgen Kuballa^c^, and Bernd Nidetzky^a,b,^*

^a^  Austrian Centre of Industrial Biotechnology, Krenngasse 37, A-8010 Graz, Austria

^b^ Institute of Biotechnology and Biochemical Engineering, Graz University of Technology, NAWI Graz, Petersgasse 12, A-8010 Graz, Austria

^c^ GALAB Laboratories GmbH, Am Schleusengraben 7, D-21029 Hamburg, Germany

*Corresponding author: Bernd Nidetzky, e-mail: bernd.nidetzky@tugraz.at, phone: +43 316 873 8400

**ADDITIONAL METHODS**

**Cloning of CSS and SiaT into vector systems pBICI and pDUAL**

Plasmid construction of pDUAL and pBICI vector backbones was described previously (1). Genes of CSS from *N. meningitidis* (2) and a three-residue N-terminal extended α2,3-SiaT from *P. dagmatis* (3) were integrated into the co-expression plasmids. Primers were purchased from Integrated DNA Technologies, Inc. (IA, USA) and listed in Table 1. Parts were assembled by overlap extension PCR (OExPCR, (4) and restriction cloning. *E. coli* Top10F’ was used for plasmid amplification and plasmids were extracted using GeneJET Plasmid Miniprep Kit (Thermo Fisher Scientific Inc., Waltham, MA, USA). Plasmids were sequence-verified by restriction analysis and sequencing (Microsynth Austria GmbH, Austria).

**Assays for *N*-acetyl neuraminic acid lyase and β-galactosidase activity in *E. coli* BL21 (DE3) cells**

β-Galactosidase activity in induced *E. coli* BL21 (DE3) was determined in disrupted cells (20 mg_protein_/ml). Ortho-nitrophenyl-β-galactoside (oNPG, 1.0 mM) was mixed with 0.2 µg of disrupted cells in 100 µl Tris/HCl (100 mM, pH 8.0) and the reaction was monitored over 10 min in 96-well plates on a multi-mode microplate reader (37°C, 405 nm, FLUOstar® Omega, BMG LABTECH GmbH). Deionized water was used as a blank.

*N*-acetyl-neuraminic acid aldolase activity of *E. coli* BL21 (DE3) was determined by incubation of permeabilized cells (0.013 mg_cwm_/ml) in 0.1 M Tris/HCl (pH 8.0) containing 20 mM CTP, 20 mM lactose, 20 mM Neu5Ac, 40 mM MgCl_2_ and 0.2 mM L-Cys in a total volume of 1 ml. The reaction was performed at 37°C and 450 rpm over 2 h. Samples were taken for carbohydrate measurement on HPLC as described in the main text.

**Thin layer chromatography (TLC)**

Conversion reactions with the cell catalysts were analyzed with TLC for fast, semi-quantitative assessment before quantification with HPLC. Samples from the reaction (20 µl) were stopped by heat inactivation (99°C, 15 min) and kept on ice for 15 min. Part of this sample (1 µl) was then spotted on TLC Silica gel 60 F_254_ plates (Merck, Darmstadt, Germany) and analytes were separated with 1-butanol:acetic acid:H_2_O (2:1:1, v/v). After completion of the separation, TLC plates were dried and treated with a staining solution contained 0.5% thymol (w/v) in ethanol and sulfuric acid (97%) (95:5 % w/w).

**Storage and reaction stability for CSS and α2,3-SiaT in permeabilized cells**

Enzyme stability in the permeabilized cells was determined with the pH shift assay described in the main text. The final concentration of cell wet mass in the reaction mixture for the pH shift assay was 0.26 mg/ml.

For testing of storage stability permeabilized cells stored at -20°C were thawed and added to the reaction mixture for the pH shift assay. Storage stability was monitored once a week over several weeks.

For monitoring of enzyme stability under reaction conditions permeabilized cells were diluted to a cell wet mass of 10 mg/ml and incubated (at least in triplicate) at reaction conditions (25/37°C; 450 rpm 100 mM Tris/HCl; pH 8.0) on an Eppendorf Thermomixer comfort. Sample were taken at different times (0, 0.5, 1, 2, 3, 4 and 24 h).

**ADDITIONAL TABLES**

Table S1: List of strains, genes and plasmids used in this study.

|  | Description | Reference or source |
| --- | --- | --- |
| **Strains** |  |  |
| *E. coli* BL21 (DE3) | *F^–^ompT gal dcm lon hsdS_B_ (r_B_^–^ m_B_^–^) λ(DE3 [lacI lacUV5-T7 gene 1 ind1 sam7 nin5])* | Thermo Fisher Scientific |
| *E. coli* Top10F’ | *mcrA, Δ(mrr-hsdRMS-mcrBC), Phi80lacZ(del)M15, ΔlacX74, deoR, recA1, araD139, Δ(ara-leu)7697, galU, galK, rpsL(SmR), endA1, nupG* | Invitrogen |
| **Genes** |  |  |
| α2,3-SiaT | α2,3-sialyltransferase from *Pasteurella dagmatis* | (3) |
| CSS | CMP-Neu5Ac synthetase from *Neisseria meningitidis* | (2) |
| **Plasmids** |  |  |
| pBICI Weak RBS | pBR322 *ori, bom,* AmpR, T7_lacO,_ weak RBS | (1) |
| pBICI Strong RBS | pBR322 *ori, bom,* AmpR, T7_lacO,_ strong RBS | (1) |
| pDUAL P_tac_ | pBR322 *ori, bom,* AmpR, T7_lacO,_ tacI | (1) |
| pDUAL P_T5_ | pBR322 *ori, bom,* AmpR, T7_lacO,_ T5 | (1) |
| pBICI_weak_ α2,3-SiaT_CSS (pBICI_weak) | pBICI Weak RBS containing CSS and α2,3-SiaT | This study |
| pBICI_strong_ α2,3-SiaT_CSS (pBICI_strong) | pBICI Strong RBS containing CSS and α2,3-SiaT | This study |
| pDUAL_tacI_ α2,3-SiaT_CSS (pDUAL_tacI) | pDUAL P_tac_ containing CSS and α2,3-SiaT | This study |
| pDUAL_T5_ α2,3-SiaT_CSS (pDUAL_T5) | pDUAL P_T5_ containing CSS and α2,3-SiaT | This study |

Table S2: CSS and α2,3-SiaT activities in whole-cells and cell-free extract of cell catalysts pBICI_weak and pBICI_strong

| Cell catalyst | Whole-cells | | Cell-free extract | |
| --- | --- | --- | --- | --- |
|  | CSS activity  U/mg_cdm_ | α2,3-SiaT activity  U/mg_cdm_ | CSS activity  U/mg_CFE_ | α2,3-SiaT activity U/mg_CFE_ |
| pBICI_weak | 570 | 555 | 340 | 588 |
| pBICI_strong | 1950 | 580 | 1590 | 530 |

Table S3:Enzyme parameters of CSS and α2,3-SiaT

| enzyme | specific activity  [U/mg protein] | *K*_M_  [mM] | *K*_eq_  [L mol^-1^] |
| --- | --- | --- | --- |
| CSS | 36 | Neu5Ac:  0.11^a^ CTP:  0.05^a^ | -^b^ |
| α2,3-SiaT | transfer: 5.7  donor hydrolysis^c^:  1.1^d^  sialidase:  0.02^d^ | CMP-Neu5Ac:  1.1^d^  Lactose:  1.5^d^ | 161.7^e^ |

Note: CSS (CMP-sialic acid synthetase from *N. meningitidis*); α2,3-SiaT (α2,3-sialyltransferase from *P. dagmatis*)
^a^ (5,6)

^b^ Reaction assumed to be quasi-irreversible

^c^ Measured in absence of acceptor

^d^ (3,7)

^e^ *K*_eq_ estimated from model fits (2)

Table S4: Oligonucleotide sequences used in OExPCR for cloning of pBICI co-expression plasmids.

| construct | primer name: sequence (5´ → 3´) |
| --- | --- |
| pBICI_weak | AatII-PdSTfw:  ttaagaaggagaGACGTCatgaaaacaatcacaatctatttagatcctgcttcattaccc  weak RBS-PdSTrev: tGGTACCGTCCTGTGTGATGTACAtcagtggtggtggtggtggtgc  weak RBS-CSSfw: cactgaTGTACATCACACAGGACGGTACCatgagaggatcgcatcaccatcacc  SalI-CSSrev:  ttgGTCGACTTATTCCTTGTGATTAAGAATGTTTTCTGCCTGTTG  SalI-T7TTfw: TAAGTCGACcaaaaaacccctcaagacccgtttagaggc  AatII-PT7rev: tgtgattgttttcatGACGTCtctccttcttaaagttaaacaaCattatttctagagggg |
| pBICI_strong | AatII-PdSTfw: ttaagaaggagaGACGTCatgaaaacaatcacaatctatttagatcctgcttcattaccc  strong RBS-PdSTrev: GGTACCtttctcctcttTTGTACAtcagtggtggtggtggtggtgc  strong RBS-CSSfw: cactgaTGTACAAaagaggagaaaGGTACCatgagaggatcgcatcaccatcacc  SalI-CSSrev: ttgGTCGACTTATTCCTTGTGATTAAGAATGTTTTCTGCCTGTTG  SalI-T7TTfw: TAAGTCGACcaaaaaacccctcaagacccgtttagaggc  AatII-PT7rev: tgtgattgttttcatGACGTCtctccttcttaaagttaaacaaCattatttctagagggg |

Table S5: Oligonucleotide sequences used in OExPCR for cloning of pDUAL co-expression plasmids.

| construct | Name: sequence (5´ → 3´) |
| --- | --- |
| pDUAL_T5 | KpnI-CSSfw: AgaatctaaagaggagaaaGGTACCatgagaggatcgcatcaccatcacc lt0-SalI-CSSrev: CAGGAGTCGTCGACTTATTCCTTGTGATTAAGAATGTTTTCTGCCTGTTG  SalI-lambdat0fw: ACAAGGAATAAGTCGACGACTCCTGTTGATAGATCCAGTAATGACCTCAG  Bsp1407I-T7TTfw: ccaccaccaccaccactgaTGTACActagcataaccccttggggcc  AatII-PdSTfw: ttaagaaggagaGACGTCatgaaaacaatcacaatctatttagatcctgcttcattaccc PdSTrev: tcagtggtggtggtggtgg  AatII-originfw: ttgttttcatGACGTCtctccttcttaaagttaaacaaaattatttctagaggggaattg KpnI-PT5rev: ctcatGGTACCtttctcctctttagattcTGTGTGAAATTGTTATCCGCTCAC |
| pDUAL_tacI  (All primers identical to pDUAL_T5 besides KpnIPT5rev) | KpnI-Ptacrev:  ctcatGGTACCtttctcctctttagattccattatacgagccgatgatta |

**MODEL EQUATIONS**

Reactions for CSS and α2,3-SiaT are described by Michaelis-Menten parameter (*V*_max_, *K*_M_) mass action (Γ) kinetics, as shown in Eq. (S1-S5).

The mass balance for all modelled reactants is shown in Eq. S6 - S11. Note that Eq. S6, S9 and S10 include terms for the hydrolysis of CMP-Neu5Ac by α2,3-SiaT (1-*R*_h_, Eq. S9 and S10) which releases Neu5Ac (*R*_h_, Eq. S6). Note that hydrolysis can happen at the levels of binary complex (enzyme and CMP-Neu5Ac) and ternary complex (enzyme, CMP-Neu5Ac and lactose) of the α2,3-SiaT. The level of the binary complex is determined by the degree of saturation of CMP-Neu5Ac-bound enzyme with lactose (parameter *F* in Eq. S2 and S3). The hydrolysis at the level of binary complex is expressed by Eq. S3. Hydrolysis at the ternary complex is expressed with the parameter *R*_h_ that accounts for a true “error” hydrolysis catalyzed by the enzyme, as demonstrated in earlier work (ref. 7). The total hydrolysis of of CMP-Neu5Ac is the sum of the contributions of conversion of binary and ternary complex, as shown in Eq. S6.

*R*_h_ is the ratio of hydrolysis and transfer (for CMP-Neu5Ac). *R*_h_ is assumed invariant with the concentration of lactose (2).

CMP-Neu5Ac consumption (Eq. S8) and CMP production (Eq. S11) are independent of *R*_h_.

**Michaelis-Menten mass action kinetics**

$$\begin{aligned} V\left( CSS \right)=\frac{V_{\max\left( CSS \right)}\left[ CSS \right]\left[ Neu5Ac \right]\left[ CTP \right]}{\left( K_{M\left( Neu5Ac \right)}+\left[ Neu5Ac \right] \right)\left( K_{M\left( CTP \right)}+\left[ CTP \right] \right)}\#Eq.\left( S1 \right) \end{aligned}$$

$$\begin{aligned} V_{t}\left( \mathrm{SiaT} \right)=\frac{V_{\max\left( SiaT\_transfer \right)}\left[ SiaT \right]\left[ CMPNeu5Ac \right]}{\left( K_{M\left( CMPNeu5Ac \right)}+\left[ CMPNeu5Ac \right] \right)}F\left( 1-\frac{\Gamma_{\left( \mathrm{SiaT} \right)}}{K_{eq\left( \mathrm{SiaT} \right)}} \right)\#Eq.\left( S2 \right) \end{aligned}$$

$$\begin{aligned} V_{h}\left( \mathrm{SiaT} \right)=\frac{V_{\max\left( SiaT\_donor\_hydroylsis \right)}\left[ SiaT \right]\left[ CMPNeu5Ac \right]}{\left( K_{M\left( CMPNeu5Ac \right)}+\left[ CMPNeu5Ac \right] \right)}\left( 1-F \right)\#Eq.\left( S3 \right) \end{aligned}$$

$$\begin{aligned} F_{\left( \mathrm{SiaT} \right)}=\frac{\left[ Lactose \right]}{K_{M(Lactose)}+\left[ Lactose \right]}\#Eq.\left( S4 \right) \end{aligned}$$

$$\begin{aligned} \Gamma_{\left( \mathrm{SiaT} \right)}=\frac{\left[ 3SL \right]\left[ CMP \right]}{\left[ CMPNeu5Ac \right]\left[ Lactose \right]}\#Eq.\left( S5 \right) \end{aligned}$$

**Cascade reaction**

$$\begin{aligned} \frac{d\left[ Neu5Ac \right]}{dt}=-V\left( CSS \right)+V_{t}\left( \mathrm{SiaT} \right)R_{h}+V_{h}\left( \mathrm{SiaT} \right)\#Eq.\left( S6 \right) \end{aligned}$$

$$\begin{aligned} \frac{d\left[ CTP \right]}{dt}=-V\left( CSS \right)\#Eq.\left( S7 \right) \end{aligned}$$

$$\begin{aligned} \frac{d\left[ CMPNeu5Ac \right]}{dt}=V\left( CSS \right)-V_{t}\left( \mathrm{SiaT} \right)-V_{h}\left( \mathrm{SiaT} \right)\#Eq.\left( S8 \right) \end{aligned}$$

$$\begin{aligned} \frac{d\left[ Lactose \right]}{dt}=-V_{t}\left( \mathrm{SiaT} \right)\left( 1-R_{h} \right)\#Eq.\left( S9 \right) \end{aligned}$$

$$\begin{aligned} \frac{d\left[ 3SL \right]}{dt}=V_{t}\left( \mathrm{SiaT} \right)\left( 1-R_{h} \right)\#Eq.\left( S10 \right) \end{aligned}$$

$$\begin{aligned} \frac{d\left[ CMP \right]}{dt}=V_{t}\left( \mathrm{SiaT} \right)+V_{h}\left( \mathrm{SiaT} \right)\#Eq.\left( S11 \right) \end{aligned}$$

**ADDITIONAL FIGURES**


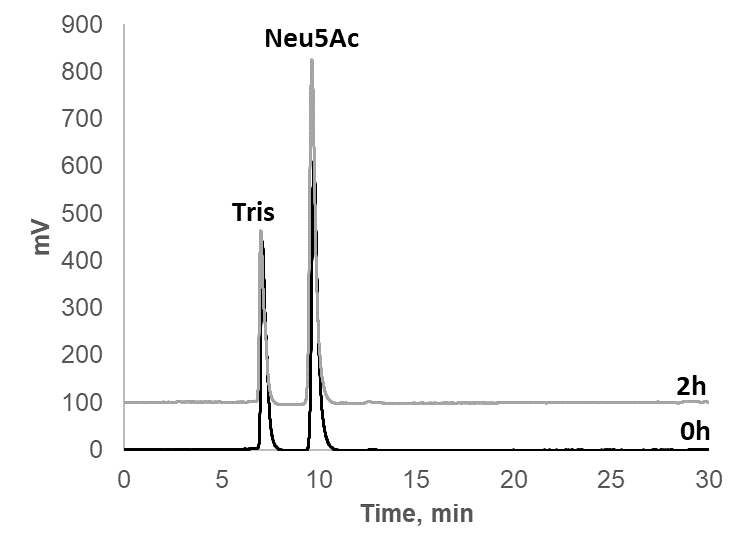


Figure S1: Test of background activity by NAL. HPLC measurement of Neu5Ac (20 mM) incubated with IPTG-induced (25°C, 21 h) empty-pBICI vector *E. coli* BL21(DE3) (1.0 mg_cwm_/ml) in 100 mM Tris/HCl (pH 8.0) over 2 h to observe ManNAc and pyruvate formation by NAL activity. Retention times: 7.1 min (Tris), 9.7 min (Neu5Ac); 11.2 min (pyruvate), 13.2 min (ManNAc). No activity (Neu5Ac consumption; ManNAc and pyruvate release) was detected.


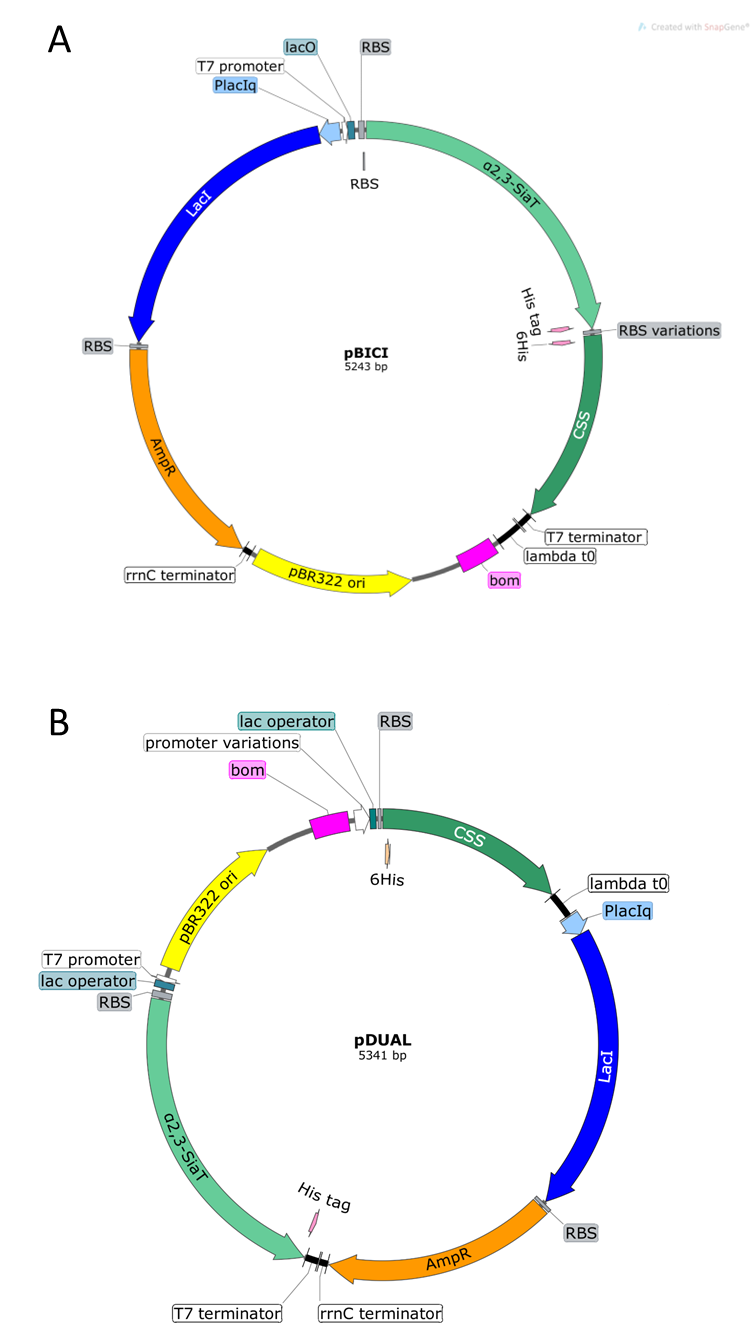


**Figure S2**: Vector maps of pBICI and pDUAL. The backbone consists of a medium-copy number origin of replication (pBR322 *ori*) in combination with a bom site, different transcription terminators (T7, lambda t0, *rrnC*), a regulator for the T7 promoter containing *lacO* site (LacI), a constitutive mutant promoter of lacI with increased strength (PlacIq) and an ampicillin resistance marker (AmpR) (1). (A) Bicistronic expression vector (pBICI): The strong T7_lacO_ phage promoter controls the expression of CSS and α2,3-SiaT. CSS is either expressed with a strong RBS (pBICI_strong) or a weak RBS (pBICI_weak). (B) Monocistronic expression vector (pDUAL): α2,3-SiaT is expressed with a T7_lacO_ phage promoter, while CSS expression is controlled by different promoter variants (tacI, T5). Both enzymes are equipped with a non-cleavable hexahistidine tag at the N- and C-terminus of CSS and α2,3-SiaT, respectively.

**
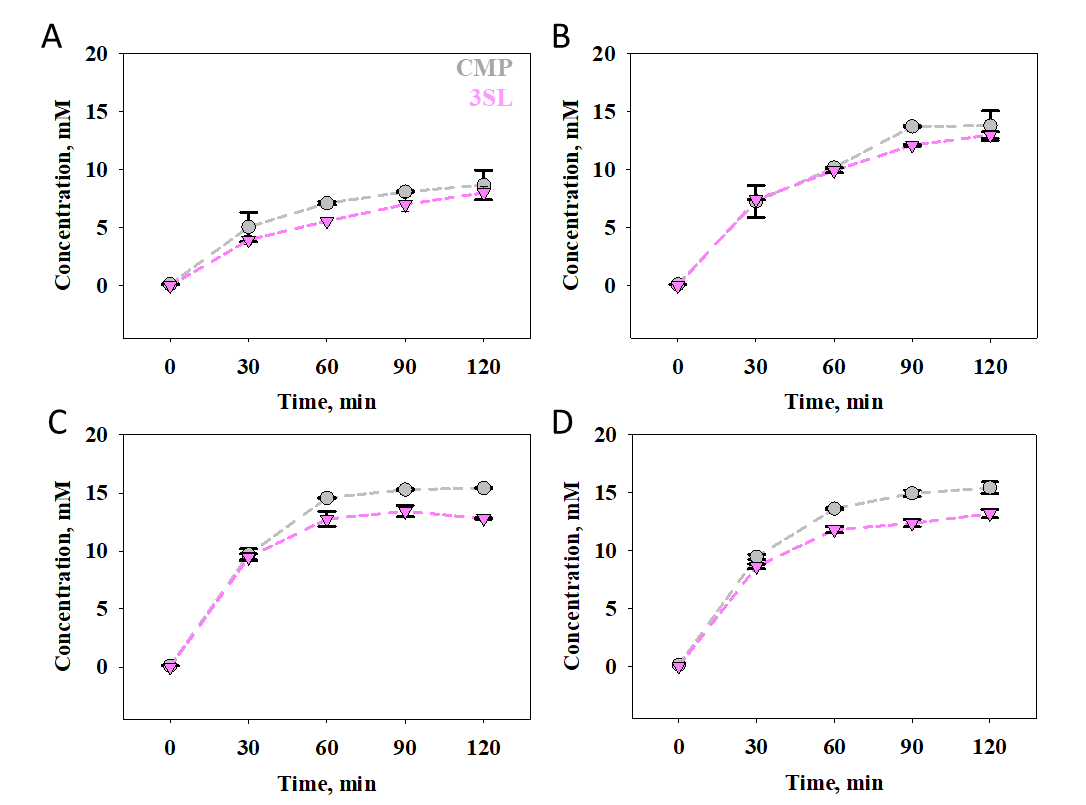
**

**Figure S3**: Comparison of the released CMP and 3SL during whole cell-catalyzed 3SL synthesis. The reactions are performed with ~20 mM of each Neu5Ac, CTP and lactose at 37°C and pH 8.0. The CSS activity is normalized to 0.40 U/ml in each reaction based on variable loading of the cell catalyst, hence also activity ratio (*R*_E_) of CSS:α2,3-SiaT, as follows. **A**: 0.22 g_cdm_/L, pBICI_strong, *R*_E_ 1:0.3; **B**: 0.58 g_cdm_/L, pBICI_weak, *R*_E_ 1:1; **C**: 0.94 g_cdm_/L, pDUAL_tacI, *R*_E_ 1:3; **D**: 0.98 g_cdm_/L, pDUAL_T5, *R*_E_ 1:2). Symbols show the mean values of 3 replicate experiments and error bars show the S.D.


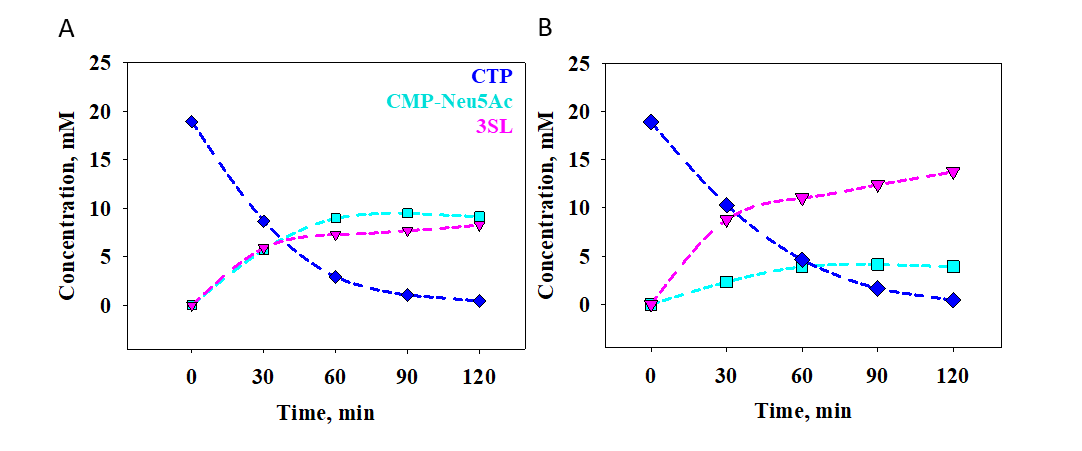


**Figure S4**: Effect of reaction temperature on the 3SL synthesis by the cell catalyst. Time course of CMP-Neu5Ac (cyan square), CTP (blue diamond) and 3SL (pink triangle) levels in a conversion reaction (~20 mM Neu5Ac, CTP and lactose, 100 mM Tris/HCl, pH 8.0). Cell concentrations were adjusted based on CSS activity of cells (determined at the reaction temperature) to reach a CTP consumption rate of 0.4 mM/min in each conversion. Cell catalyst pBICI_strong was used for synthesis at reaction temperature of 37°C (enzymatic ratio CSS:SiaT = 1:0.5; 0.14 g_cdm_/L; A) and 25°C (enzymatic ratio CSS:SiaT = 1:3; 0.31 g_cdm_/L; B).

**REFERENCES**

1. Schwaiger KN, Voit A, Dobiašová H, Luley C, Wiltschi B, Nidetzky B. Plasmid design for tunable two-enzyme co-expression promotes whole-cell production of cellobiose. Biotechnol J. 2020;2000063:1–10.

2. Schelch S, Eibinger M, Gross Belduma S, Petschacher B, Kuballa J, Nidetzky B. Engineering analysis of multienzyme cascade reactions for 3ʹ‐sialyllactose synthesis. Biotechnol Bioeng. 2021;118(11):4290–4304.

3. Schmölzer K, Ribitsch D, Czabany T, Luley-Goedl C, Kokot D, Lyskowski A, et al. Characterization of a multifunctional α2,3-sialyltransferase from *Pasteurella dagmatis*. Glycobiology. 2013;23(11):1293–304.

4. Horton RM, Hunt HD, Ho SN, Pullen JK, Pease LR. Engineering hybrid genes without the use of restriction enzymes: gene splicing by overlap extension. Gene. 1989;77(1):61–8.

5. Gilbert M, Watson DC, Wakarchuk WW. Purification and characterization of the recombinant CMP-sialic acid synthetase from *Neisseria meningitidis*. Biotechnol Lett. 1997;19(5):417–20.

6. He N, Yi D, Fessner WD. Flexibility of substrate binding of cytosine-5’-monophosphate-*N*- acetylneuraminate synthetase (CMP-sialate synthetase) from *Neisseria meningitidis*: An enabling catalyst for the synthesis of neo-sialoconjugates. Adv Synth Catal. 2011;353(13):2384–98.

7. Schmölzer K, Eibinger M, Nidetzky B. Active-site His85 of *Pasteurella dagmatis* sialyltransferase facilitates productive sialyl transfer and so prevents futile hydrolysis of CMP-Neu5Ac. ChemBioChem. 2017;18(15):1544–50.
